# Using a Novel Absolute Ontogenetic Age Determination Technique to Calculate the Timing of Tooth Eruption in the Saber-Toothed Cat, *Smilodon fatalis*

**DOI:** 10.1371/journal.pone.0129847

**Published:** 2015-07-01

**Authors:** M. Aleksander Wysocki, Robert S. Feranec, Zhijie Jack Tseng, Christopher S. Bjornsson

**Affiliations:** 1 Department of Biological Sciences, Clemson University, Clemson, South Carolina, United States of America; 2 Research and Collections Division, New York State Museum, Albany, New York, United States of America; 3 Division of Paleontology, American Museum of Natural History, New York, New York, United States of America; 4 Neural Stem Cell Institute, Rensselaer, New York, United States of America; NYIT College of Osteopathic Medicine, UNITED STATES

## Abstract

Despite the superb fossil record of the saber-toothed cat, *Smilodon fatalis*, ontogenetic age determination for this and other ancient species remains a challenge. The present study utilizes a new technique, a combination of data from stable oxygen isotope analyses and micro-computed tomography, to establish the eruption rate for the permanent upper canines in *Smilodon fatalis*. The results imply an eruption rate of 6.0 millimeters per month, which is similar to a previously published average enamel growth rate of the *S*. *fatalis* upper canines (5.8 millimeters per month). Utilizing the upper canine growth rate, the upper canine eruption rate, and a previously published tooth replacement sequence, this study calculates absolute ontogenetic age ranges of tooth development and eruption in *S*. *fatalis*. The timing of tooth eruption is compared between *S*. *fatalis* and several extant conical-toothed felids, such as the African lion (*Panthera leo*). Results suggest that the permanent dentition of *S*. *fatalis*, except for the upper canines, was fully erupted by 14 to 22 months, and that the upper canines finished erupting at about 34 to 41 months. Based on these developmental age calculations, *S*. *fatalis* individuals less than 4 to 7 months of age were not typically preserved at Rancho La Brea. On the whole, *S*. *fatalis* appears to have had delayed dental development compared to dental development in similar-sized extant felids. This technique for absolute ontogenetic age determination can be replicated in other ancient species, including non-saber-toothed taxa, as long as the timing of growth initiation and growth rate can be determined for a specific feature, such as a tooth, and that growth period overlaps with the development of the other features under investigation.

## Introduction

Timing of development is critical in vertebrate ecology and evolution [1–6]. Changes in the timing of life history events can have major effects on fitness and changes in the timing of development of morphological features (i.e., heterochrony) can yield major changes to the vertebrate bauplan [1–4, 7]. For ancient species, determining the relative sequence of developmental events is feasible provided that a taxon has a well preserved ontogenetic sequence of individuals; whereas identifying the absolute ontogenetic age of development for particular characteristics is much more challenging. Absolute, as opposed to relative, ontogenetic age for ancient species is often estimated based on the timing of development in modern analogs, such as age determinations for American mastodon (*Mammut americanum*) and mammoth (*Mammuthus* sp.) based on extant elephant (*Loxodonta* sp.) tooth wear stages [8]. To fully understand how taxa develop, function within ecosystems and evolve, it is essential to understand when features develop in absolute ontogenetic time.

One unique morphological feature that has evolved independently numerous times within the theriodont lineage (the clade composed of gorgonopsians and eutheriodontians, the latter including Mammalia), is the saber-tooth morphology of permanent upper canines [9–10]. This feature is most renowned in the machairodontine (Felidae) species *Smilodon fatalis*, but has evolved several times in therapsids, including within the gorgonopsids, creodonts, nimravids, barbourofelids, marsupials, and as mentioned above, the felids. The exceptional fossil record of *S*. *fatalis* at Rancho La Brea (southern California) has facilitated numerous investigations and revealed many aspects of saber-tooth morphology as well as the behavior, life history and evolution of this species [9–17]. For example, a meticulous study of numerous specimens from Rancho La Brea documented the relative tooth-eruption sequence, which provides an understanding of *S*. *fatalis* tooth eruption and cranial growth, as well as further clues to *S*. *fatalis* life history and behavior [13]. Despite the tremendous fossil record of this species, particularly at Rancho La Brea, ontogenetic age determination of the numerous individuals has remained a challenge. Based on the concept that species of comparable skeletal dimensions share similar growth rates, estimates of ontogenetic age have been assigned to stages of dental development in another machairodontine, *Homotherium serum*, utilizing the extant *Panthera leo* as a modern analog [18]. These ontogenetic age estimates are supported by stable oxygen isotope analyses that indicate similar permanent upper canine (C^1^) enamel growth rates for *H*. *serum* and *P*. *leo* [19]. However, the C^1^ enamel growth rate for *S*. *fatalis* is faster than the C^1^ enamel growth rates of *H*. *serum* and *P*. *leo* [19]. Furthermore, *S*. *fatalis* differs from *P*. *leo* in aspects of the relative timing of tooth development, such as the timing of permanent carnassial eruption relative to permanent incisor eruption, which makes *P*. *leo* less suitable as a modern analog for ontogenetic age estimates of *S*. *fatalis* than for ontogenetic age estimates of *H*. *serum* [13, 20].

This study is focused on determining the timing of developmental events in *S*. *fatalis*, with an emphasis on understanding the ontogenetic age at which individual *S*. *fatalis* had fully erupted upper canines. A fully erupted permanent dentition is vital for many carnivore species because it impacts the capacity to acquire prey (e.g., extant conical-toothed felids ordinarily dispatch their prey via a bite that employs the canines), as well as the ability to process and defend carcasses [21–22]. In particular, fully erupted C^1^s are likely to have been advantageous for *S*. *fatalis* when it came to prey acquisition [10, 14–15, 17, 23].

## Materials and Methods

### Absolute Ontogenetic Age Calculation

To calculate absolute ontogenetic ages for the development of particular morphological features in ancient species, it is necessary to find a structure (e.g., a tooth) that has a growth period that overlaps the development of the features of interest, and to have data on the timing of growth initiation and the growth rate of the key feature. It is possible to use C^1^ growth rate to calculate the absolute ontogenetic ages of initiation and conclusion of eruption for nearly all of the permanent teeth of *S*. *fatalis* because the eruption of these teeth occurs while the C^1^ forms in this species [13]. What is more, the C^1^ growth rate can be used to calculate the absolute ontogenetic timing of other coinciding developmental events (e.g., cranial suture closures and loss of most of the deciduous dentition).

Determining the absolute ontogenetic age at which C^1^ growth began is necessary for calculating the absolute ontogenetic ages of development for particular features of *S*. *fatalis*. Because the C^1^ growth appears to have begun prior to birth [24], a conservative estimate for the duration of gestation in *S*. *fatalis* can be used to estimate the absolute ontogenetic timing of C^1^ growth initiation. In mammals, gestation is correlated with body mass and the duration of gestation tends to be rather consistent within carnivoran families [21, 25]. The gestation periods of the extant large felids *Panthera tigris* (104 to 106 days), *P*. *leo* (110 days), *P*. *onca* (101 days), *P*. *pardus* (88 to 112 days), *P*. *uncia* (90 to 103 days), *Puma concolor* (82 to 96 days), and *Acinonyx jubatus* (90 to 95 days), exemplify this consistency [26–32]. The hyaenids, *Hyaena hyaena*, *H*. *brunnea*, and *Crocuta crocuta*, have periods of gestation that average 91 days, 97 days, and 110 days, respectively [33–36]. The gestation periods for large viverrids are 60 to 81 days for *Civettictis civetta* and approximately 91 days for *Arctictis binturong* [21, 37–38]. Considering that gestation is associated with body mass and that the extant large felids and other feliforms exhibit relatively similar gestation periods, it seems unlikely that the gestation period of *S*. *fatalis* deviated drastically from those observed in the extant large felids (Table 1) [21, 25]. Because the estimated body mass of *S*. *fatalis* (approximately 160 to 280 kg) [39] is most similar to the body mass of *P*. *tigris* [26, 29], *P*. *tigris* appears to be the best modern analog for *S*. *fatalis* gestation.

**Table 1:** Body Mass and Gestation of Feliforms.

Species	Body Mass (kg)	Gestation (days)
*Panthera tigris*	75 to 306	104 to 106
*Panthera leo*	110 to 225	110
*Panthera onca*	41.4 to 104.5	101
*Panthera pardus*	20 to 90	88 to 112
*Panthera uncia*	25 to 75	90 to 103
*Puma concolor*	35 to 65	82 to 96
*Acinonyx jubatus*	35.0 to 40.2	90 to 95
*Lynx canadensis*	4.5 to 17.3	63 to 70
*Lynx rufus*	4.1 to 18.3	50 to 70
*Felis silvestris catus*	3.0 to 3.4	63
*Crocuta crocuta*	40.5 to 69.2	110
*Hyaena brunnea*	28.0 to 47.5	97
*Hyaena hyaena*	25 to 55	90 to 92
*Arctictis binturong*	11 to 32	91.5
*Civettictis civetta*	7 to 20	60 to 81

Data compiled from [26–38, 40–44].

Studies have generally shown that deciduous tooth mineralization and permanent tooth mineralization do not begin during the early stages of gestation [45–49]. For example, in the small felid, *Felis silvestris catus*, tooth buds begin developing at about 28 to 32 days of its 63 day gestation period [29, 46]. Similarly, calcified tooth germs are not perceptible in fetuses of the rodent *Myocaster coypus* before 100 to 105 days of its 125 day gestation period [50]. Given that approximately 1cm of the forming C^1^ crown is present in *S*. *fatalis* at about the time of birth [24] and given that the average C^1^ enamel growth rate is 5.8mm/month [19], it appears likely that the C^1^ of *S*. *fatalis* began to mineralize roughly one month prior to birth. Utilizing one month prior to birth as the point of initial C^1^ growth, absolute ontogenetic age ranges were calculated for developmental events using a C^1^ enamel growth rate range of 5 to 7.5 mm/month determined previously through examination of stable oxygen isotope values incorporated into tooth enamel [19, 51] and C^1^ crown lengths previously assigned to particular *S*. *fatalis* developmental events [13] (see S1 Text).

Although it is possible to assign specific values to the timing of developmental events, ontogenetic age ranges are more appropriate for describing the timing of developmental events in the life history of *S*. *fatalis*. Tooth development and the fusion of cranial sutures are known to occur at various ontogenetic ages for different individuals of other felid species due to factors such as sexual dimorphism [20, 52]. For instance, the timing of initial permanent tooth eruption in *P*. *leo* differs by as much as 6 weeks between males and females [53]. In *S*. *fatalis*, studies suggest that there is either no sexual dimorphism in skull size or that the degree of size sexual dimorphism in *S*. *fatalis* is less than that observed in *P*. *leo* [54–56]. However, *S*. *fatalis* skulls do appear to exhibit a degree of shape variation that is equivalent to the degree of morphometric sexual dimorphism in *Panthera* [56]. The exact nature of *S*. *fatalis* sexual dimorphism notwithstanding, the timing of developmental events in the life histories of mammalian species can be influenced by other differences that exist between individuals, such as disparities in nutritional health that alter the rate of cranial suture fusion [57]. Thus, even though there may be a popular desire to bestow fixed ontogenetic ages to the developmental events in the life history of *S*. *fatalis*, we believe that calculated ontogenetic age ranges serve as more appropriate guidelines.

### C^1^ Eruption Rate Calculation

**Fig 1:**
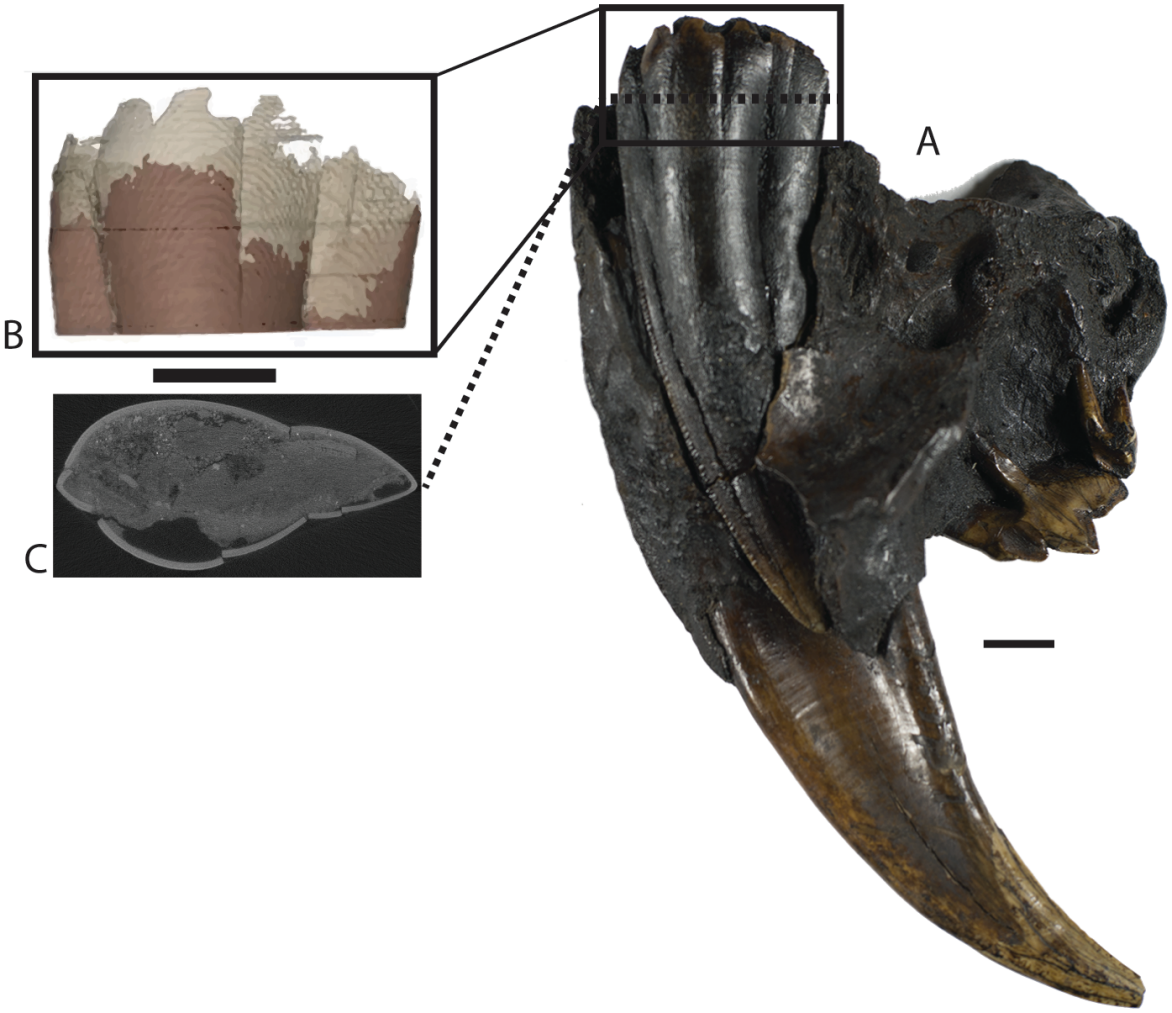
*Smilodon fatalis* specimen UCMP 152565 with partially formed permanent upper canine. (A) Photograph of UCMP 152565. Scale bar represents 1 cm. (B) Image of the 3D model created from the uCT data of UCMP 152565. The model represents the proximal-most portion of the canine. Dark brown color indicates completely mineralized enamel, whereas light brown is unfinished or unmineralized portion of canine. Scale bar represents 1cm. (C) A slice from the uCT data of UCMP 152565.

In *S*. *fatalis*, C^1^ enamel formation was completed before the loss of the dC^1^ and the completion of C^1^ eruption. Thus, another approach, beyond using the canine growth rate calculated using stable oxygen isotopes [19], was necessary to determine the absolute ontogenetic ages for these two developmental events, and that was the calculation of the C^1^ eruption rate. It should be noted that C^1^ growth rate and C^1^ eruption rate are closely connected processes, but dissimilar from one another in that C^1^ growth rate describes the rate of tooth formation, whereas C^1^ eruption rate describes the rate at which the distal-most portion of the C^1^ moves in relation to its alveolar border. It was not possible to calculate the C^1^ eruption rate by merely measuring the C^1^ length in specimens of different ontogenetic stages. Preservation of the un-mineralized portion of this tooth can be inconsistent between specimens. In addition, because the formation of tooth enamel is a two-part process consisting of matrix formation and subsequent mineralization that affects stable oxygen isotope values [58], C^1^ eruption rate calculation using the previously published C^1^ growth rate [19] should be more accurate if based on C^1^ mineralized enamel lengths (S1 Text). Therefore, calculation of the C^1^ eruption rate required determination of the proximodistal lengths of C^1^ mineralized enamel, which were determined ultimately by using micro-computed tomography (μCT) to examine the proximal portion of the C^1^ of each specimen (see Fig 1).

Although the number of identified specimens and number of individuals of *S*. *fatalis* at Rancho La Brea is quite large, certain criteria (e.g., C^1^ of different ontogenetic stages) were needed to calculate the C^1^ eruption rate, which severely limited the number of specimens that were relevant for analysis. ZJT and RSF identified a total of six specimens (LACM A-3748, LACM A3775, LACMHC 2001–9, LACMHC 2003-R-1, UCMP 152565, and UCMP 152566) as suitable for C^1^ eruption rate calculation on the basis that each of these specimens has at least one intact, erupting C^1^. Due to the spatial constraints of the μCT bore, the large size of the specimens, and similarity in ontogenetic stage between some specimens, only three specimens (LACM A-3748, UCMP 152565, and UCMP 152566) were able to be scanned. For this study, a SCANCO Medical Viva CT40 μCT scanner was utilized at the Center for Biotechnology and Interdisciplinary Studies at Rensselaer Polytechnic Institute (Troy, NY). Specimens were scanned at an energy of 70kVp, an intensity of 114 uA, and a 199 ms integration time. Scanning was performed near the proximal portion of the C^1^ with voxel size of 19 μm. The resulting image stacks had maximum dimensions of 2048 x 2048 pixels. 2100 slices were produced for UCMP 152565, which was scanned in two parts. 3036 slices were produced for UCMP 152566, and 5039 slices were produced for LACM A-3748. Three-dimensional reconstructions of enamel and dentin were created using Mimics version 13.1. Because of the spatial constraints of the μCT, two specimens (UCMP 152565 and UCMP 152566) yielded data (S1 and S2 Datasets) that were sufficient for creating three-dimensional reconstructions usable in this study. LACM A-3748 was able to be scanned; however the data were incomplete because the specimen was too large to fit properly within the μCT bore, thereby making it impossible to generate a complete three-dimensional reconstruction for LACM A-3748 (see S3 Dataset).

Density differences in the form of intensity grayscale values (with fully mineralized enamel appearing white, and dentin and other bones appearing as darker shades of gray) were utilized to segment the dentin versus enamel structures in the μCT data. As a consequence of limitations in computing resources, the reconstructions were built from reduced datasets that were 50% of original resolution in the x and y dimensions, and 10–30% in the z direction. The reductions resulted in an effective x-y pixel size of 38 μm and interslice distance of 57–190 μm, providing a maximum error of +/-190 μm (i.e., the exact location of the proximal borders of dentin and mineralized enamel are not resolved below the interslice distance of the reduced dataset) in the placement of measurement points.

To determine the C^1^ mineralized enamel length of each specimen, the distance from the most proximal border of preserved dentin to the most proximal border of mineralized enamel along the anterior edge of the C^1^ was subtracted from the overall C^1^ length. Measurements of the distance between the most proximal border of preserved dentin and the most proximal border of mineralized enamel along the anterior edge of the C^1^ were made within the 3D window in Mimics to the nearest 0.01 mm. Digital calipers were used to measure the overall C^1^ length of each specimen, as well as the length of erupted C^1^ from the alveolar border. The alveolar border, defined as an anteroposterior straight line across the alveolus on the maxilla and premaxilla at its distal-most point, represented 0.0 mm. Using the previously published average C^1^ enamel growth rate of 5.8 mm/month (range = 5 to 7.5 mm/month) [19, 51], we estimated *S*. *fatalis* C^1^ eruption rate, utilizing the following equation:

$${\text{C}}^{\text{1}}\text{Eruption Rate}=\left({\text{Erupted C}}^{\text{1}}\text{Length of Specimen B}-{\text{Erupted C}}^{\text{1}}\text{Length of Specimen A}\right)/\left[\left({\text{C}}^{\text{1}}\text{Mineralized Enamel Length of Specimen B}-{\text{C}}^{\text{1}}\text{Mineralized Enamel Length of Specimen A}\right)/\left({\text{Average C}}^{\text{1}}\text{Enamel Growth Rate}\right)\right]$$ (1)

The C^1^ mineralized enamel lengths of two specimens (UCMP 152565 and UCMP 152566) were used to calculate the C^1^ eruption rate. The C^1^ eruption rate and associated lengths of erupted C^1^ (which are known via the previously published tooth replacement sequence of Tejada-Flores and Shaw (1984) [13]) were used to determine the period of time (in months) from initial C^1^ eruption to the loss of the dC^1^, as well as the period of time from initial C^1^ eruption to the completion of C^1^ eruption. The period of time from initial C^1^ eruption to the loss of the dC^1^ was added to the absolute ontogenetic age at which the C^1^ begin to erupt (calculated using the C^1^ enamel growth rate range of 5–7.5mm/month [19]) in order to calculate the absolute ontogenetic age range of dC^1^ loss. Similarly, the period of time from initial C^1^ eruption to the completion of C^1^ eruption was added to the absolute ontogenetic age at which the C^1^ begin to erupt in order to calculate the absolute ontogenetic age range for the completion of C^1^ eruption.

## Results

**Fig 2:**
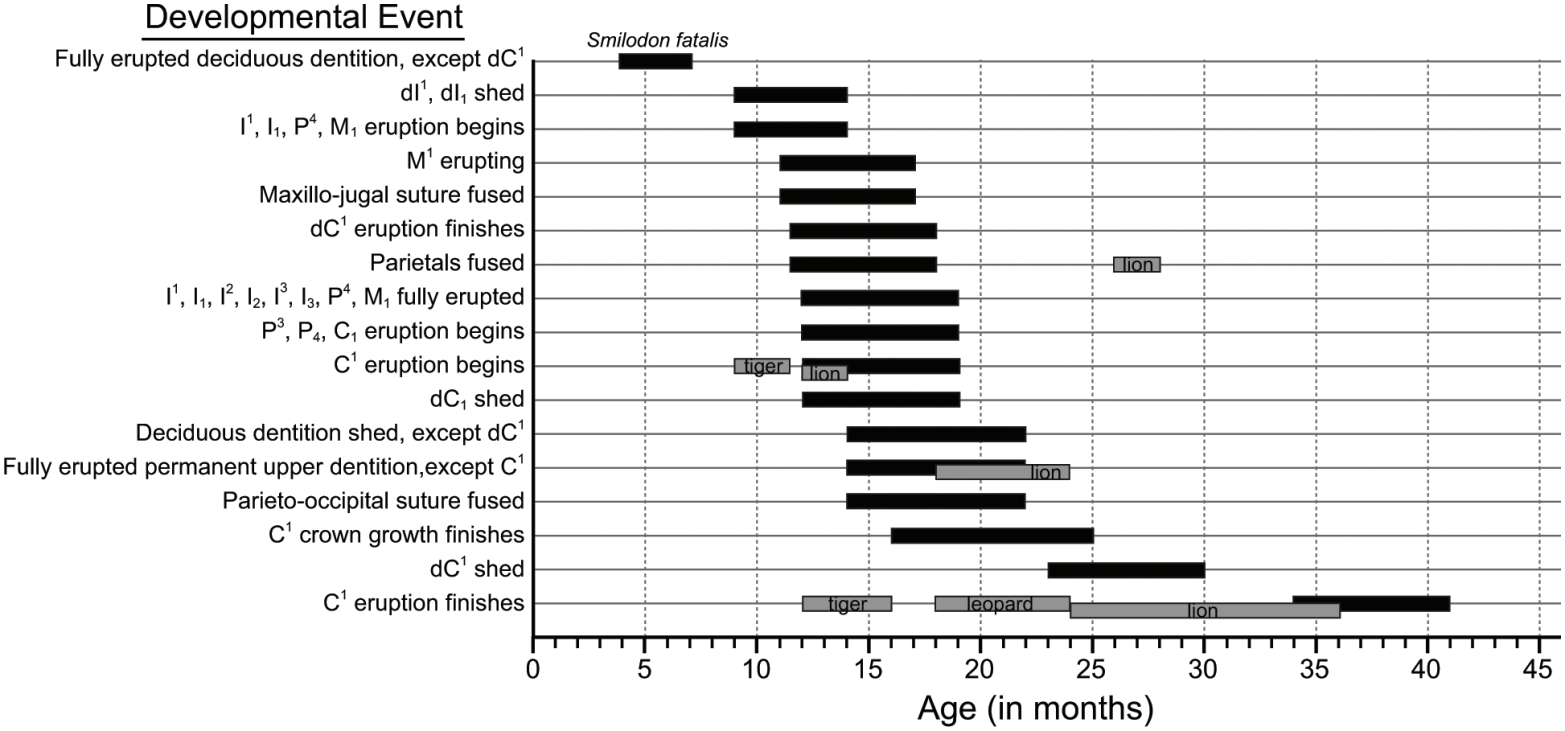
Absolute ontogenetic age ranges for developmental events. Extant conical-toothed felid data from [20, 26, 52, 59].

Utilizing the absolute ontogenetic age of C^1^ growth initiation, the C^1^ growth rate range previously calculated from stable oxygen isotope values [19] and the previously determined lengths of C^1^ associated with particular developmental stages [13], absolute ontogenetic age ranges were calculated for a number of *S*. *fatalis* developmental events (Fig 2). With the exception of dC^1^, absolute ontogenetic age range determination was not feasible for individual deciduous teeth because *S*. *fatalis* specimens with erupting deciduous dentition, aside from the dC^1^, are scarce in the fossil record [13]. Based on the data available, the deciduous dentition of *S*. *fatalis*, except for the dC^1^, was fully erupted by approximately 4 to 7 months of age, and the dC^1^ finished erupting between 11.5 to 18 months of age. The first deciduous teeth to be shed were the deciduous upper and lower first incisors (dI^1^ and dI_1_) at approximately 9 to 14 months of age. During this same interval, the permanent dentition begins to erupt with the emergence of the permanent upper and lower first incisors, as well as the upper and lower carnassials (I^1^, I_1_, P^4^ and M_1_). By the age of 11 to 17 months, the small permanent upper molars (M^1^) were in the process of erupting and the maxillo-jugal suture was fused. The parietals fused by 11.5 to 18 months old. The permanent upper and lower first, second and third incisors, as well as the carnassials (I^1^, I_1_, I^2^, I_2_, I^3^, I_3_, P^4^ and M_1_) were fully erupted at 12 to 19 months of age. The permanent upper third premolars, lower fourth premolars, and upper and lower canines (P^3^, P_4_, C^1^, and C_1_) began to erupt at the age of 12 to 19 months. Over this same interval, the dC_1_s were shed. By the age of 14 to 22 months, the dC^1^s were the only remaining deciduous teeth. The permanent dentition, with the exception of the C^1^s, was complete at 14 to 22 months old. Also, the parieto-occipital suture was fused by 14 to 22 months of age. The C^1^ crowns finished growing between 16 and 25 months old.

The μCT data from UCMP 152565 and UCMP 152566 revealed mineralized enamel lengths of 80.4mm and 98.6mm, respectively. The developing C^1^ of UCMP 152565 had not yet erupted past the alveolar border and the distal-most portion is 5.4mm above that border. The C^1^ of UCMP 152566 had traversed the alveolar border and the distal-most portion is 13.3mm below that border. Using Eq 1, the C^1^ eruption rate was calculated to be 6.0mm/month, which is similar to the average C^1^ enamel growth rate previously calculated from stable oxygen isotope values (5.8mm/month [19]). As reported above, the absolute ontogenetic age calculations based on the average C^1^ enamel growth rate reveal that the C^1^ began to erupt at the age of 12 to 19 months. Using the C^1^ eruption rate, the additional time from initial C^1^ eruption until the loss of the dC^1^s was calculated to have been approximately 11 months, which implies that the dC^1^s were shed at about 23 to 30 months. Also, based on the C^1^ eruption rate, it appears that a period of nearly 22 months was required for the C^1^s to fully erupt, which suggests that the C^1^s finished erupting at roughly 34 to 41 months of age.

## Discussion

### Species Comparison

Determining the timing of tooth development and eruption in *S*. *fatalis* makes it possible to compare aspects of the life history of this saber-toothed species to those of several extant conical-toothed species. The data suggest that dC^1^ eruption, and thus eruption of the deciduous dentition, in *S*. *fatalis* did not conclude until 11.5 to 18 months of age. In contrast, the entire deciduous dentition is completely erupted at about 1.5 to 2 months in *P*. *tigris* and by 2 to 3 months in *P*. *leo* [20, 26]. *S*. *fatalis* and *P*. *leo* are also dissimilar in that the parietals appear to have fused at 11.5 to 18 months in *S*. *fatalis* as opposed to 26 to 28 months in *P*. *leo* [20]. Tejada-Flores & Shaw (1984) [13] point out that the closure of this suture and the growing sagittal crest in *S*. *fatalis* indicate that this relatively young age class possessed a large attachment area for the temporalis muscles. Permanent tooth eruption began at a slightly later age in *S*. *fatalis* (9–14 mo.) compared with many of the extant conical-toothed species such as *P*. *concolor* (8 mo.), *P*. *leo* (8-9 mo.), and *P*. *tigris* (8.5-9.5 mo.) [20, 26, 27]. Conversely, permanent carnassial eruption finished at a younger age in *S*. *fatalis* (12–19 mo.) than it does in *P*. *leo* (18–24 mo.) [20]. Notably, permanent carnassial eruption coincides with permanent incisor eruption in *S*. *fatalis*, whereas permanent carnassial eruption takes place after permanent incisor eruption in *P*. *leo* [13]. Nevertheless, on the whole, *S*. *fatalis* appears to have had delayed dental development compared to dental development in similar-sized extant felids [20, 26]. Another one of the more distinctive features of *S*. *fatalis* tooth development is that the dC^1^s appear to have been retained until approximately 23 to 30 months of age, which suggests that *S*. *fatalis* possess both the erupted dC^1^s and the erupting C^1^s for a period of about 11 months. This period of time is greater than those observed in several extant conical-toothed felids (e.g., *P*. *leo* possess both the erupted dC^1^s and the erupting C^1^s for a period of about 3 months) [20, 27, 52, 53, 59–62]. Considering that the upper canines of saber-toothed carnivores seem to have been at a greater risk of fracture from medio-laterally directed forces than are the conical upper canines of extant felids [63], this relatively prolonged period of time that the deciduous upper canines remain in place alongside the erupting permanent upper canines may be noteworthy. Further exploration is currently underway to evaluate the possibility that this pattern of canine replacement (i.e., deciduous canines remaining in position alongside permanent canines for a large percentage of the permanent canine eruption process) functions as a protective mechanism that reduces the likelihood of saber-tooth fracture.

### Behavioral and Ecological Implications

Although the *S*. *fatalis* fossil record at Rancho La Brea is extensive, there is a scarcity of *S*. *fatalis* specimens from younger age classes, which Tejada-Flores and Shaw (1984) suggest is not merely the result of the poor preservation of more fragile juveniles. Explanations for this scarcity include parental behavior that kept cubs in close proximity to den sites and that preferred den sites were not common near Rancho La Brea [13]. *P*. *leo* cubs become somewhat mobile at approximately 4 to 6 weeks old, which is also the approximate age at which they are introduced to the rest of pride [53]. Similarly, *P*. *tigris* cubs tend to remain at den sites until they become fairly mobile at 1 to 2 months old and *P*. *onca* cubs do not leave den sites until about 2 months of age [28, 59, 64]. Our results suggest that *S*. *fatalis* individuals of less than 4 to 7 months of age were not frequently preserved at Rancho La Brea, and are consistent with the ideas that den sites were rarely located near Rancho La Brea and that parental behavior restricted the cubs to the den sites [13]. The results also seem to be consistent with the concept that there may have been a prolonged period of parental care upon consideration that it appears *S*. *fatalis* juveniles did not possess fully erupted deciduous upper canines until 11.5 to 18 months of age. Watts et al. (2009) [65] showed that for an extant dietary specialist, the bone-cracking *C*. *crocuta*, the protracted development of the specialized feeding apparatus affects the rapid feeding performance of juveniles not only on carcasses containing bone, but also on softer foods, which represents a handicap to feeding in general and makes maternal aid crucial to juvenile survival while the specialized feeding apparatus is still developing. In a similar way, the relatively delayed completion of deciduous upper canine eruption in *S*. *fatalis* could have impacted hunting in juveniles by rendering them less effective as predators prior to possessing their deciduous upper canines, which could have made *S*. *fatalis* juveniles more reliant on parental support while the deciduous upper canines were still developing. In addition to the explanations that parental care restricted the cubs to the den sites and that den sites were rarely located near Rancho La Brea [13], the dearth of *S*. *fatalis* specimens from younger age classes may be due to predator avoidance behavior that typically prevented *S*. *fatalis* cubs from leaving cover to directly feed on carcasses mired in the asphalt seeps. *P*. *tigris* cubs are known to be present at kill sites by as early as 3 months of age, but the cubs remain concealed within cover while tigresses make the kills alone; subsequently tigresses lead the cubs to the kill sites [59, 64]. On other occasions, tigresses transfer the kills, meeting their cubs at alternate locations [59]. Similarly, *P*. *leo* adults exhibit the behavior of moving kills to their cubs and *P*. *leo* adults sometimes move kills into cover while they are in the presence of other predators [53]. Given that there is a relative abundance of carnivores preserved at Rancho La Brea that has been attributed to predators becoming trapped in the asphalt seeps as they attempted to feed on the carcasses of mired animals, the asphalt seeps may have attracted an array of predators, potentially making it an especially hazardous area for young *S*. *fatalis* cubs to move out of cover [23, 66–73]. Thus, it appears plausible that infrequent den site selection near Rancho La Brea, parental behavior that restricted cubs to the den sites and predator avoidance behavior that prevented cubs from directly visiting carcasses mired within the asphalt seeps all contributed to the infrequent preservation of *S*. *fatalis* individuals from younger age classes.

### Saber-Tooth Development

The data, as expected, suggest that the upper canines of *S*. *fatalis* finished erupting at an older absolute ontogenetic age (34–41 mo.) than the absolute ontogenetic age at which they finish erupting in *P*. *leo* (24–36 mo. [20]). Yet, the difference in these absolute ontogenetic ages at which the C^1^s finish erupting is not as different as one might expect if predictions were made on the basis of C^1^ crown heights alone (43–52 mm in *P*. *leo* vs. ~130 mm in *S*. *fatalis*) [13, 30]. The *S*. *fatalis* C^1^ crown growth rate (5.8 mm/mo.) was faster than that of *P*. *leo* (2.9 mm/mo.)[19] and the data from this study suggest that the C^1^ eruption rate of *S*. *fatalis* was faster than the C^1^ eruption rate of *P*. *leo* [20, 30]. It is apparent, however, that C^1^ growth rates are not uniform for all saber-toothed carnivores. The C^1^ growth rates for *Smilodon* (5.8 mm/mo. for *S*. *fatalis* and 6.3 mm/mo. for *Smilodon gracilis*) are faster than the C^1^ growth rate of the scimitar-toothed sabercat, *H*. *serum* (2.8 mm/mo.) [19, 51, 74]. What is more, there are marked distinctions in the types of tooth morphology [75, 76] and tooth growth exhibited by saber-toothed carnivores (e.g., unlike the C^1^s of *S*. *fatalis*, the C^1^s of *Thylacosmilu*s *atrox* appear to have been hypselodont [77]). Despite these differences, the evolution of saber-teeth may be based upon similar evolutionary changes. In particular, it is thought that regulation of the dental epithelial stem cell niche might have the evolutionary flexibility to produce variation in tooth shape, growth rates, and timing of root development [78, 79]. The loss of fibroblast growth factor 10 (Fgf10) signaling appears to be associated with a shift from crown formation to root development; a delay in this change from crown formation to root formation could lead to the development of hypsodont teeth and the absence of this transition to root formation could generate hypselodont teeth [78, 80]. Therefore, future research may reveal that despite the substantial differences in tooth crown height, growth rate, and growth duration among the various saber-toothed and conical-toothed carnivores, the evolution of saber-teeth may involve similar changes to dental epithelial stem cell niche regulation.

This study emphasized the calculation of absolute ontogenetic age for the development of the dentition of *S*. *fatalis*, but this technique for absolute ontogenetic age determination should be applicable in some other species. In particular, if a starting ontogenetic age (i.e., initiation of growth) and growth rate can be calculated for a particular morphological feature, it is possible to calculate its absolute ontogenetic age of completion and the absolute ontogenetic ages of any features that develop simultaneously. Although, hypothetically, it is possible to construct absolute ontogenetic age estimates via linking together multiple developmental processes (e.g., the sequential growth periods of several different teeth), we suspect that approach may prove to be less accurate. Absolute ontogenetic age estimates contingent on a linked series of developmental processes are likely subject to a greater degree of inaccuracy because each additional developmental process includes another range of values due to the variation that exists between individuals of a given species. The technique utilized in this study (focusing on C^1^ growth and eruption) may not be useful for absolute ontogenetic age determination in all ancient species, as C^1^ growth and eruption may not coincide with the majority of tooth developmental events, but it would seem that studies on taxa with tusks (e.g., proboscideans, walruses, and narwhals) would yield accurate ontogenetic age determinations akin to this study. Rountrey et al. (2012) proposed a similar methodology for aging young woolly mammoths (*Mammuthus primigenius*) [81]. Another useful technique for ontogenetic age determination that has been used to study ancient species, including *S*. *fatalis*, is the analysis of pulp cavity closure using radiographs of mandibles and lower canines [55, 82, 83]. It appears that this technique was difficult to apply to *S*. *fatalis* because this saber-toothed felid has a relatively reduced lower canine, whereas the technique was more effective for studying the conical-toothed felid, *Panthera atrox* [55]. All in all, absolute ontogenetic age determinations should be possible within taxa that preserve anatomical features that record incremental growth, and whose growth trajectories overlap with those of other structures.

## Conclusions

Based on our results, *Smilodon fatalis* individuals of less than 4 to 7 months old were not frequently fossilized at Rancho La Brea. It appears that during development *S*. *fatalis* possessed both the erupted dC^1^s and the erupting C^1^s for roughly 11 months and that the C^1^s of *S*. *fatalis* ultimately finished erupting at approximately 34 to 41 months of age. Despite the widespread interest in this particular life history event, it would be prudent to consider that future analysis of additional specimens may facilitate the refinement of the C^1^ eruption rate and enhance absolute ontogenetic age determination accuracy for the developmental events that occur subsequent to C^1^ crown growth completion (i.e., the absolute ontogenetic age that the dC^1^s are shed and the absolute ontogenetic age that eruption of the C^1^s completed). Comparison with the tooth development of the extant conical-toothed felid *P*. *leo* suggests that *S*. *fatalis* required more time to develop its complete permanent dentition, but less additional time than would be expected if predictions were based solely on C^1^ crown heights; an outcome that is attributable, at least in part, to the relatively faster C^1^ growth and eruption rates of *S*. *fatalis*. The data from μCT analyses reveal a C^1^ eruption rate that is comparable to the previously identified average C^1^ enamel growth rate. Overall, this new technique provides a strategy by which it is possible to determine the absolute ontogenetic age of extinct individuals of some species. Although the present study focuses primarily on tooth development, it also demonstrates that it is possible to ascertain the absolute ontogenetic age of other developmental events (e.g., the fusion of cranial sutures). Analysis of juvenile and sub-adult specimens that possess associated postcranial material and an intact C^1^ would present the opportunity to obtain many additional insights regarding *S*. *fatalis* development and life history.

## Supporting Information

S1 Text C^1^ Growth Rate Calculation from Stable Oxygen Isotopes. (DOCX)

S1 Dataset μCT data from UCMP 152565 (Complete scan). 3D model available at doi:10.7910/DVN/29450.
(ZIP)

S2 Dataset μCT data from UCMP 152566 (Complete scan). 3D model available at doi:10.7910/DVN/29450.
(ZIP)

S3 Dataset μCT data from LACM A–3748 (Incomplete scan). 3D model available at doi:10.7910/DVN/29450.
(ZIP)
