# Supplementary material for: Using a Novel Absolute Ontogenetic Age Determination Technique to Calculate the Timing of Tooth Eruption in the Saber-Toothed Cat, Smilodon fatalis
Source: PLoS One. 2015 Jul 1;10(7):e0129847. doi: 10.1371/journal.pone.0129847 (PMC4489498; doi:10.1371/journal.pone.0129847)
Supplement: S1 Text — (DOCX) [file pone.0129847.s001.docx]

**S1 Text. C^1^** **Growth Rate Calculation from Stable Oxygen Isotopes**

Stable oxygen isotope values that are incorporated into the tooth enamel of animals can be used to calculate the growth rate of the enamel. Oxygen isotope results are expressed in the standard δ‑notation: δ^18^O = [(^18^O/^16^O_sample_/^18^O/^16^O_standard_) - 1] × 1000. δ^18^O values are reported relative to the V‑PDB or SMOW standard. Oxygen isotopes in mammalian enamel depends on the isotopic composition of ingested water, fractionation of oxygen isotopes between tooth enamel and body water, and the metabolism of the particular animal (Land et al., 1980; Longinelli, 1984; Luz et al., 1984; Luz and Kolodny, 1985; Koch et al., 1989; Kohn, 1996; Kohn et al., 1996, 1998; Kohn and Welker, 2005). Mammalian predators ingest water from two sources; the meteoric water that they drink and the food that they consume. For larger mammals, such as *S. fatalis*, the ingested meteoric water has a greater influence on the isotope value in tooth enamel than the water from consumed food. Meteoric water is affected by climatic influences such as temperature and humidity, such that δ^18^O values are more positive where and when it is warmer (e.g., summer) and more negative where and when it is colder (e.g., winter) (Dansgaard et al., 1982; Rozanski et al., 1992; Fricke and O’Neil, 1996; Balasse et al., 2003). An animal that drinks meteoric water in the same general area and whose teeth grow over the course of a year will display a positive and negative cycle in enamel δ^18^O values (Fricke and O’Neil, 1996; Balasse et al., 2003; Cerling and Sharp 1996). Yet, the cycle does not necessarily reflect the absolute variation of δ^18^O values ingested because oxygen isotopes get incorporated into the tooth during a two-part process: matrix formation and mineralization (Passey and Cerling, 2002). The δ^18^O cycle is dampened because the two processes generally occur at different times. However, this research is concerned with the δ^18^O pattern archived within the tooth enamel, not the actual δ^18^O values ingested. Because the isotope cycle reflects isotope values across seasons, the length of tooth enamel for a whole cycle, (positive peak to positive peak) reflects one year of growth. Therefore, the length of tooth enamel can be divided by the amount of time represented (i.e., 12 months) in order to calculate the growth rate.

**SI References**

Land LS, Lundelius EL, Valastro S. Isotopic ecology of deer bones. Palaeogeography, Palaeoclimatology, Palaeoecology. 1980;32: 143-151.

Longinelli A. Oxygen isotopes in mammal bone phosphate: A new tool for paleohydrological and paleoclimatological research? Geochimica et Cosmochimica Acta. 1984;48: 385-390.

Luz B, Kolodny Y. Oxygen isotope variations in phosphate of biogenic apatites, IV. Mammal teeth and bones. Earth and Planetary Science Letters. 1985;75: 29-36.

Luz B, Kolodny Y, Horowitz M. Fractionation of oxygen isotopes between mammalian bone-phosphate and environmental drinking water. Geochimica et Cosmochimica Acta. 1984;48: 1689-1693.

Koch PL, Fisher DC, Dettman D. Oxygen isotope variation in the tusks of extinct proboscideans: A measure of season of death and seasonality. Geology. 1989;17: 515-519.

Kohn MJ. Predicting animal δ^18^O: Accounting for diet and physiological adaptation. Geochimica et Cosmochimica Acta. 1996;60: 4811-4829.

Kohn MJ, Schoeninger MJ, Valley JW. Herbivore tooth oxygen isotope compositions: Effect of diet and physiology. Geochimica et Cosmochimica Acta. 1996;60: 3889‑3896.

Kohn MJ, Schoeninger MJ, Valley JW. Variability on oxygen isotope compositions of herbivore teeth: Reflections of seasonality or developmental physiology. Chemical Geology. 1998;152: 97-112.

Kohn MJ, Welker JM. On the temperature correlation of δ^18^O in modern precipitation: Earth and Planetary Science Letters. 2005;231: 87–96.

Dansgaard W, Clausen HB, Gundestrup N, Hammer CU, Johnson SF, Kristinsdottir PM, et al. A new Greenland deep ice core: Science. 1982;218: 1273–1277.

Fricke HC, O'Neil JR. Inter- and intra-tooth variation in the oxygen isotope composition of mammalian tooth enamel phosphate: implications for palaeoclimatological and palaeobiological research. Palaeogeography Palaeoclimatology Palaeoecology. 1996;126: 91-99.

Rozanski K, Araguas‑Araguas L, Gonfantani R. Relation between long-term trends of Oxygen-18 isotope composition of precipitation and climate: Science. 1992;258: 981–985.

Balasse M, Smith AB, Ambrose SH, Leigh SR. Determining sheep birth seasonality by analysis of tooth enamel oxygen isotope ratios: the Late Stone Age site of Kasteelberg (South Africa). Journal of Archaeological Science. 2003;30: 205-215.

Cerling TE, Sharp ZD. Stable carbon and oxygen isotope analysis of fossil tooth enamel using laser ablation. Palaeogeography, Palaeoclimatology, Palaeoecology. 1996;126: 173-186.

Passey BH, Cerling TE. Tooth enamel mineralization in ungulates: Implications for recovering a primary isotopic time-series: Geochimica et Cosmochimica Acta. 2002;66: 3225‑234.
